# The Influence of Built Environment Factors on Elderly Pedestrian Road Safety in Cities: The Experience of Madrid

**DOI:** 10.3390/ijerph19042280

**Published:** 2022-02-17

**Authors:** Daniel Gálvez-Pérez, Begoña Guirao, Armando Ortuño, Luis Picado-Santos

**Affiliations:** 1 Departamento de Ingeniería del Transporte, Territorio y Urbanismo, Escuela Técnica Superior de Ingenieros de Caminos, Canales y Puertos, Universidad Politécnica de Madrid, Calle del Profesor Aranguren, 3, 28040 Madrid, Spain; begona.guirao@upm.es; 2 Escuela Politécnica Superior, Universidad de Alicante, 03690 San Vicente del Raspeig, Spain; arorpa@ua.es; 3 CERIS, Instituto Superior Técnico, Universidade de Lisboa, Av. Rovisco Pais, 1049-001 Lisboa, Portugal; luispicadosantos@tecnico.ulisboa.pt

**Keywords:** elderly pedestrians, road safety, road traffic collisions, accident analysis, built environment, street design

## Abstract

With the progressive ageing of the population, the study of the relations between road safety and elderly users is becoming increasingly relevant. Although the decline of pedestrian skills in the elderly has been widely studied in the literature, few studies have been devoted to the contributing built environmental factors of the elderly pedestrian collisions, such as the sidewalk density, the presence of traffic lights, or even some indicator related to land use or the socioeconomic features of the urban fabric. This paper contributes to the limited literature on elderly pedestrian safety by applying a negative binomial regression to a set of built environmental variables to study the occurrence of accidents involving elderly and younger (non-elderly) pedestrians in Madrid (Spain) between 2006 and 2018. The model considers a selection of built environmental factors per city district, linked to land use, infrastructure, and socioeconomic indicators. Results have highlighted that the elderly pedestrian collisions could be avoided with the existence of a wider sidewalk in the district and a greater traffic lights density. Unlike younger pedestrian accidents, these accidents are much more favored in ageing districts with higher traffic flows.

## 1. Introduction

An increase in life expectancy has led to a generalized phenomenon of population ageing. The United Nations forecasts that global life expectancy will increase from 70 years in 2015 to 83 years by 2095 [1]. This demographic change means, unavoidably, more elderly are actively part of mobility and road traffic, and this fact is translated into an increasing number of elderly road fatalities and severe injuries. Sustainable cities should also be inclusive for the elderly pedestrians, and mobility (mainly walking) is a fundamental prerequisite for the well-being of older persons [2,3,4]. Promoting walking for elderly pedestrians in sustainable cities implies the analysis of the urban built environment variables that affect the occurrence of elderly pedestrian accidents, because in most of the cases, policymakers and other professionals (e.g., urban planners, urban designers, and architects) could implement actions on the built environment variables.

Although elderly mobility has more advantages than drawbacks, in terms of road safety, population ageing shows an indirect negative effect as the number of elderly road fatalities and severe injuries is increasing [5]. For the coming years, forecasts at European level are not better: while at the moment one road traffic fatality out of five is aged 65 or over, it is expected that by 2050 one road traffic fatality out of three will be an older person if the risk rates of older people and younger age groups decline at the same pace [5]. Crashes involving users over 65 years old will rise to alarming figures if nationwide policy actions are not taken to improve safety. Moreover, elderly pedestrians and cyclists are the weakest group as, within the entire European population, the elderly make up 39% of all pedestrian fatalities and 40% of all pedal cyclist fatalities compared to 18% and 19% of all car driver and passenger fatalities [5]. In OECD countries, persons 65 years and older represent 13% to 20% of the population, but they make up more than 50% of pedestrian fatalities [6]. Urban areas are especially risky scenarios for the elderly road safety as there are remarkably more elderly fatalities (55%) than there are middle-aged fatalities (33%) in urban areas.

The higher road accident risk of the elderly has been often linked to the reduction in physical and mental faculties with advancing age, which leads to inappropriate (and unexpected) behavior in elderly pedestrians and drivers. A large body of research [7,8,9] has dealt with specific physical and mental limitations of the elderly as road users, paying special attention to drivers [10,11]. However, while fatal accidents involving elderly drivers are still very few [12,13], elderly pedestrian fatalities in urban scenarios are statistically a matter of concern. Physical and mental limitations of elderly pedestrian are usually related to a poorer vision and hearing, lower walking speeds, longer reaction time, reduced ability to make head and neck movements, or less muscle agility. These physical and mental limitations can be exacerbated by age-related illnesses and certain chronic medication. Comorbidity (having more than on illness) is also more usual among the elderly population and is also linked to a higher crash risk [5]. Due to their physical and mental state, the elderly group also registers greater fatality rates [14].

In the literature, surveys and questionnaires to elderly road users have helped to investigate elderly pedestrians’ and drivers’ perception on road safety [8,15]. Older pedestrians and drivers can compensate with “self-regulation” actions for age-related functional declines. “Self-regulation” means that the individual is aware, acknowledges, and has insight of their functional impairments, being conscious of his own declining capacities and adapting his behavior to their limitations (e.g., avoiding complex traffic situations or only crossing at formal pedestrian crossings). Due to their mental maturity and “self-regulation”, in general, older road users are more cautious compared to younger age groups [8].

Literature on older pedestrians’ perception of their own declining capacity suggests that they have good awareness when there is clear feedback from the urban built environment. The urban built environment is very important for older pedestrians as inadequate infrastructures and unfamiliar environments can increase such anxieties and, in some cases, also reduce mobility [16,17]. For example, the fear of falling is a common fear among elderly pedestrians, and, through surveys, poorly maintained pavements were identified as the most important risk factor in their outdoor environment [18,19]. The built and physical environment can be a strong determinant of mobility, but in most studies the analysis of the built environment and walking infrastructure is reduced to subjective perceptions declared by the elderly by surveys and rarely backed by pedestrian collision data and the analysis of the accident location in the city.

In fact, little is known about the contributing built environmental factors and injury outcomes of the elderly pedestrian collisions. Apart from road safety education of elderly pedestrians, the implementation of actions on the urban built environmental (adapting sidewalks, street and intersections signaling and lighting, urban car speed reduction, etc.) is a key issue to reduce elderly pedestrian fatalities and severe injuries. Therefore, investigating the contributory urban built environment factors to collision and injury risk is a first step in the development of appropriate road safety strategies and countermeasures.

This paper contributes to the limited existing literature by studying the built environment contributory factors to elderly pedestrian collisions in the city of Madrid (Spain) for the period 2006–2018. Madrid, with a population of more than 3 million inhabitants, is the capital and biggest Spanish city. Spain has one of the lowest road fatality rates per million of inhabitants (the seventh in 2019) among European countries, but population ageing and elderly pedestrian fatalities are starting to become a matter of concern. In 2019, 19% of the Spanish population was aged over 65 and 70% of pedestrian fatalities in urban roads were individuals over 65 years old [20]. Methodology was based on a negative binomial regression, applied to a database made of built environmental factors and urban accidents involving one vehicle and one elderly or younger (non-elderly) person, thus capturing the effect of the built environmental factor on the accident occurrence per city district (administrative division). Previous works developed in Madrid, at a lower level of data disaggregation, by Gálvez-Pérez, Guirao, and Ortuño [21] already showed the importance of the built environmental variables. However, some key ad hoc variables were missing in the study, and the modelling results obtained for elderly pedestrian collisions were not compared with the analysis of the accident experienced by the rest of younger pedestrian. For that reason, this research is not only an extension of the cited study but also a comparative analysis among two age groups with a larger number of studied built environment features, obtaining a better holistic approach of the problem.

The paper is divided into the following sections: Section 1 contains the introduction; Section 2 presents the state of the art on pedestrian road safety and elderly pedestrians as a study group; Section 3 gives a detailed description of Madrid as a case study. Section 4 explains the model structure, application to the database, and discusses the most important results affecting the process. Finally, Section 5 presents the conclusions and future research lines drawn from this research.

## 2. State of the Art on Road Safety of Pedestrians and Elderly Pedestrians

The literature has demonstrated that active mobility is more intricate than a logistics optimization problem [22,23,24,25], reflecting that walking (like cycling) is complex behavior resulting from the interaction between individuals, groups, and their environment. While frequent car routes for drivers are limited to the road network and route choice is more dependent on generalized cost optimization, pedestrians’ routes include all the city streets’ networks, and route choice does not necessarily follow the shortest itinerary in distance or time or that more efficient in terms of energy or cost, as it usually depends on other route features [26]. As a consequence, pedestrian mobility analysis requires integrated perspectives and approaches from different disciplines such as urbanism, psychology, engineering, ecology, and physical health.

The complexity of the pedestrian mobility analysis is also extended to the study of the pedestrians´ road safety. Pedestrian fatalities and injuries due to road safety accidents are some of the limited negative externalities derived from walking in our cities. The location of the accident can be considered a built environment factor [27]. According to Stocker et al. [27], road safety risk in the built environment can be studied at regional level (population density, land use mix, urban sprawl, socioeconomic variables, etc.) and at local level (pedestrian infrastructure and roadway design). With this approach, conditions of the built environment include also factors such as traffic speeds, traffic flows, and visibility, and the pedestrian accident risk is a mixture of the level of risk provided by the built environment and the type of pedestrian (elderly, young children and young adults, disable pedestrians, intoxicated pedestrians, gender roles, etc.).

Tiwari [28] has studied the relationship between the evolution of pedestrian safety research and the measures to reduce these type of accidents for the last 120 years, taking as reference year the date of the first reported pedestrian fatality in 1899 [29]. Although the epidemiology of pedestrian crashes through available accident databases has been continuously developed, findings of Tiwari [28] describe an initial special focus on the analysis of pedestrian crossing behavior, and research study designs based primarily on field observations, complemented with pedestrian interviews, state preference studies and risk perception at various facilities. Street crossings were identified from the beginning as risky locations for pedestrians because there is always a time limitation to making a decision to cross the street: the time elapsing between the pedestrians first observation of the oncoming vehicle and the arrival of the vehicle at the crossing. As a consequence, gap acceptance has been one of the first variables used in pedestrian field observation, and later, preferences of route choice and location for crossing roads were analyzed. These primary field observations at road crossings have migrated in the last decades towards the use of video cameras, image processing [30], and multi-agent simulation systems [31] to analyze pedestrian behavior in general. The first age group of pedestrians investigated in detail was children [32], in order to better understand their cognitive skills and limitations to avoid road accidents.

Tiwari [28] pointed out two main results from the research progress on pedestrian road safety. The first one deals with individual gap acceptance levels, which are quite dependent of the width of the crossing points, but can be strongly mitigated by individual capabilities. Indirectly, this result accepts “self-regulation” of older pedestrians when their capabilities decline (e.g., older people, who walk slower, will take longer gaps). The second result is related to children road safety, giving priority to the improvement of the built environment over traditional pedestrian traffic education (especially for children below the age of 10 years). Despite the large body of research on pedestrian behavior and risk exposure, pedestrian crashes have not decreased at the desirable levels in both motorized and less-motorized countries. A new research approach is required to increase road safety of pedestrians, and there is a consensus on built environment principles that can lead to safer cities for pedestrians [33]. In this regard, the concern on designing a better built environment for certain population groups (such as the elderly) in urban scenarios has increased, and the idea of creating “age-friendly” cities has been promoted by institutions such as the World Health Organization [34].

Pedestrian and roadway infrastructure variables are key variables of the built environment at local level [27], and the literature shows a broad inventory of infrastructure features affecting pedestrian road safety. In the recent literature, some authors [18,35,36] have analyzed pedestrian road safety in relation to urban road type and traffic flows. The works developed by Galanis et al. [36], unlike in the case of Corazza et al. [18] and Demasi et al. [35], are more focused on pedestrian behavior in relation to urban road type and traffic flows. The main results by Galanis et al. [36], focusing on pedestrian illegal behavior, underlined that a low level of motorized traffic flow in combination with maintenance and mobility problems in pedestrian infrastructure incites pedestrians to walk outside the sidewalk (in the street) and underestimate their safety issues. In the studies developed by Corazza et al. [18], the pavement of the sidewalk has been directly related to pedestrian road safety, as distressed or too narrow sidewalks induce pedestrians to walk outside the sidewalks and on the carriageways, which is very unsafe. Demasi et al. [35] proposed a methodology to estimate the level of road safety for vulnerable users (pedestrians, cyclists, and motorcyclists) of each section of a street and the hazard index (with infrastructure variables) of the overall branch. Research developed recently by Kim [37] represents a milestone in the study of elderly pedestrian collisions, comparing this group of accidents with younger pedestrian collisions in a specific type of road section, the intersection level. This work has proved the influence of land use, ambient conditions, and intersection characteristics on pedestrian safety and showed differences for both age groups. Specifically, three-way intersections, raised medians, street trees and park and recreational land use were found to have a positive effect on the elderly pedestrians’ safety, and the number of bus stops increased the chance to have an elderly pedestrian collision. Moreover, Kim [37] demonstrated that some measures implemented to reduce pedestrian collisions may favor an age group against others. For example, according to Kim [37], intersections with crosswalks or colored crosswalks do not contribute to elderly pedestrians’ safety, but rather to the safety of younger pedestrians. Muhan Lv et al. [38] analyzed the occurrence of vehicle–elderly pedestrian collisions in relation to the characteristics of the built environment at the road segment level (microlevel) in a district of Shanghai. They considered the number of elderly pedestrian accidents in each segment as dependent variables, taxi flow and elderly population as exposure variables, and built environment features, extracted from online databases and image processing, as independent variables. The authors built both Poisson and GWPR models to assess the effect of built environment on the occurrence of elderly pedestrian collisions and found that road segments near schools, supermarkets, traditional markets, bus stops, and metro stations were more dangerous for elderly pedestrians. Moreover, it was found, through the GWPR model, that the influence of the built environment features varied throughout the studied district. For instance, green space may improve elderly pedestrian road safety only in noncongested environment.

As described in the mentioned research works, pedestrian road safety studies that have analyzed the infrastructure built environment as a safety-contributing factor rarely single out the elderly group. These studies focused mainly on pedestrian crossings [15,39,40] and sidewalk pavement state [18]. The majority of elderly pedestrian fatalities occur in crashes in which the elderly pedestrian is hit by another vehicle, and this scenario is overrepresented in such accidents in which they initiated a crossing maneuver [5] (European Commission, 2015). In relation to signalized pedestrian facilities, Koepsell et al. [39] found that a high rate of older-pedestrian crashes still occurs at signalized (or marked) pedestrian facilities, and, specifically, signalized crosswalks generate a 2.1 times increased crash risk for older pedestrians (even after controlling methodologically for confounding factors such as site characteristics and pedestrian and vehicle volumes). In general, elderly pedestrians tend to believe that the rest of road users obey traffic regulations, which might give them too much confidence in these rules, and this fact increases their crash risk [8]. Additionally, older pedestrians tend to wear dark clothes, which can reduce their visibility for drivers at intersections [8].

In relation to the sidewalk pavement state, uneven surfaces are a matter of concern especially for older pedestrians because this population group has a higher risk of falling, stumbling, or stripping while walking compared with younger adults. For the elderly, maintaining postural stability and balance is more difficult [8], and some authors have shown [41] that the rate of single-pedestrian accidents is significantly higher than the rate of any other accident type for older road users, but injuries in this type of accident tend to be less severe [8].

Built environmental factors at regional level [27] include also neighborhood design and land use and socioeconomic features of the districts where the elderly pedestrians live. Many authors have studied the relationship between pedestrian collisions and macrolevel built environmental factors [42,43,44,45]. Wedagama et al. [42] found that pedestrian casualties in the city center zone are particularly associated with an increase in retail and community land use during working hours. In the city center zone, out of working hours, an increase in retail land use (almost certainly clubs and bars) is also associated with an increase in pedestrian casualties. Wier et al. [43] considered, as a built environmental factor to explain pedestrian crashes, the proportion of people living in poverty and the proportion aged 65 and older. Ukkusuri et al. [44] found that tracts of land (districts or neighborhoods) with a greater proportion of industrial, commercial, and open land use types have greater likelihood for collisions while tracts with a higher fraction of residential land use have significantly lower likelihood of pedestrian collisions. Moreover, census districts that have a greater number of transit stops and schools are more likely to have greater pedestrian crashes. Some authors have even demonstrated that some built environmental factors contribute to pedestrian collisions more than road infrastructure conditions do. Apardian and Smirnov [45] found that socioeconomic neighborhood features are more significant to predict the occurrence of pedestrian collisions than is traffic exposure, measured in Vehicles Miles Travelled (VMT). Furthermore, they suggested that a proper policy to improve road safety in urban areas would be periodic renovation and building of easy-to-adapt environments, because traffic volume or land use changes may deteriorate the road safety of a certain territory. Sugie Lee, Junho Yoon, and Ayoung Woo [46] studied Seoul pedestrian safety in relation to pedestrian age and the price of housing in the neighborhoods on a macro-level scale. They built 12 separate negative binomial regression models to calculate the number of collisions in a neighborhood, disaggregating these crashes by the age of the pedestrian (elderly pedestrians and all pedestrians), the severity of the injury of the pedestrian (total crashes, KSI, and slight), and the price of the housing in the neighborhood (high- and low-price housing). They found that traffic regulators, such as crosswalks and four-way intersections, had a substantial impact on the occurrence of elderly crashes in areas with low housing prices. It was suggested that this result was caused by an uneven distribution of special pedestrian road safety measures. In addition, the authors remarked on the importance of collaborating in a multidisciplinary work group (engineer and non-engineer specialists) to address elderly pedestrian road safety issues through improvements in built environments and educational programs for elderly pedestrians and drivers.

This paper helps to shed light on the influence of built environmental factors on elderly pedestrian road safety. Although road safety education is still an important tool to avoid accidents, Tiwari [28], based on a literature review on pedestrian road safety, pointed out that decreasing fatalities and injuries is only possible if we take focused and targeted actions on the built environmental factors. Until now, the most recommended countermeasures [5] to protect elderly pedestrians from vehicle crashs are focused on reducing interactions between pedestrians and other road user types, and reducing the average speed of motorized traffic at locations with high pedestrian flow. There is also a need to implement measures that improve the conspicuity of pedestrians for drivers and specifically improve the perception of elderly pedestrians about other road users. These latter actions need a better understanding of elderly pedestrian crashes, in order to make decisions on the changes needed in the built environment.

These results lead us to the scientific need to complement studies based on road safety perceptions of elderly pedestrian and the mobility of elderly pedestrians with the analysis of the accident location in the urban road network. The next section describes the city of Madrid (Spain) as a case study for this research. An exploratory analysis of the elderly pedestrian accidents database (2006–2018) is presented per city district, as well of a selection of street built environment variables and socioeconomic factors.

## 3. Materials and Methods

### 3.1. Madrid Case Study

**Figure 1:**
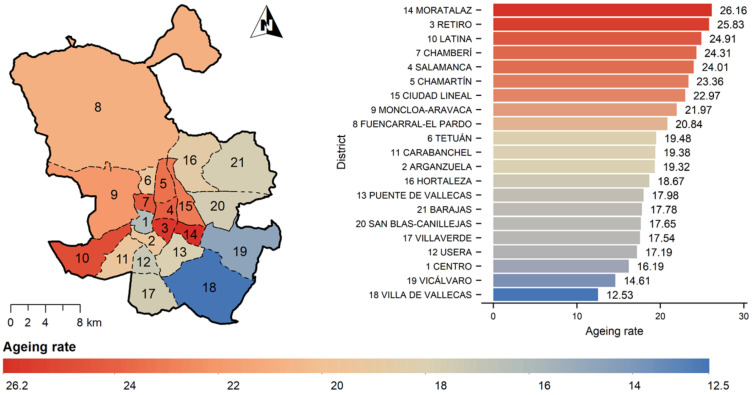
Madrid ageing rates for city districts (2018).

Spain, together with Japan, Finland, Sweden, Greece, Italy, and Germany, is among the countries in the world with a larger number of older adults. In 2019, approximately 19.3% of Spanish people were over the age of 65, and almost 6% were over 80 years old [47]; this figure could reach 40% in 2060 [48]. The city of Madrid offers a good case study for this research as it is the capital of one of the most rapidly ageing nations in the world and it also has a high proportion of elderly residents (19%). Large cities offer opportunities for better management of dedicated aging resources, and Madrid has almost 3.5 million inhabitants and is administratively divided into 21 heterogeneous districts, which are further subdivided into 131 neighborhoods (barrios). Figure 1 shows the ageing rate per city district in Madrid, revealing a higher ageing rate for central districts. In this case study, there is a great heterogeneity in terms of administrative district surface, and bigger districts are located in the periphery of the city, involving a lower residential proportion, and a lower population density and aging rate. In terms of number of inhabitants, although districts located in the periphery have low density, due to their high surface area, their population (number of inhabitants) is higher than that of central districts.

Madrid also provides a good road safety database for the pedestrian collisions study. The quality of the available accident database determines, to a large degree, the success and approach of any research on road safety. Compared to other national databases, the Spanish database is sufficiently consolidated [49,50]. The pedestrian collision data used in this research were extracted from the Spanish Accident Statistics Database and consist of accidents on Madrid city streets involving a single vehicle and a pedestrian, during a period of 13 years (2006–2018). According to the literature [5], the majority of elderly pedestrian fatalities occur in crashes in which the elderly pedestrian is hit by another vehicle. There is a higher rate of single-pedestrian accidents with older roads, but injuries in this type of accident tend to be less severe [8] with lower rates of fractures (intracranial and other injuries) compared to those who are involved in pedestrian–vehicle events [8,41]. Moreover, in the Madrid accident database, the profile of pedestrian accident involving “1 person and 1 pedestrian” was predominant in the sample (89% of vehicle–pedestrian collisions).

One of the weakest points of the Spanish accident database is the absence of traffic exposure data (traffic flow), street road layout, and traffic signaling information associated to the accident location. Moreover, the Spanish Accident Statistic Database does not supply the accident location with GPS coordinates (as in the US), using instead the kilometric point on the road of an interurban road or the closest number of a street (the name of two streets in case of accidents located in intersections) in urban scenarios, leading to further data-processing problems. Collecting these variables is very laborious, but is the only way to obtain a holistic approach for a road safety study. Furthermore, this information, if obtained, is very valuable for all stages of this line of research.

In this study, comparison between elderly pedestrian accidents and non-elderly pedestrian accidents is necessary to analyze the variables that specifically affect elderly pedestrian. Consequently, two dependent variables have been considered: the number of vehicle–elderly pedestrian collisions and the number of vehicle–non-elderly pedestrian collisions in each Madrid district. With this criteria, 20,236 records of vehicle–pedestrian collisions were filtered. Later, records of people involved in these accidents were studied to acquire collisions that have a configuration of “1 person and 1 vehicle”. Thus, 18,118 (89% of vehicle–pedestrian collisions) records were filtered to be studied. At this point, a homogenous and tidy dataset was already built, containing basic information about vehicle–pedestrian collisions in Madrid city: date, time, and type of the accident and age, gender, and injury level of the pedestrian and the driver. The last step of this procedure was to divide the ad hoc created database into two separated databases, considering the age of the pedestrian: one for pedestrians that were 65 years or older and one for the rest of the records. The result was two separate groups of data with 4663 vehicle–elderly pedestrian collisions and 13,455 vehicle–non-elderly pedestrian collisions. An initial assessment of these data highlights the fragility of the elderly age group over the rest of the population, because this age group accounts for 51% of total fatalities and 34% of total serious injuries, but only accounts for 26% of total accidents of the studied type.

**Figure 2:**
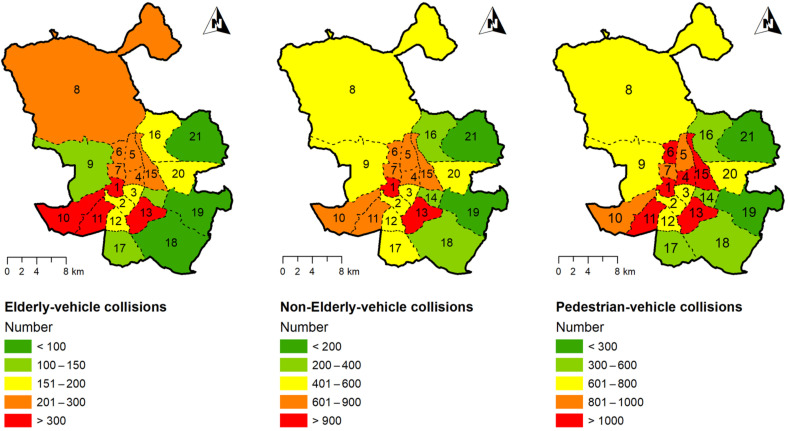
Number of collisions suffered by elderly pedestrians, non-elderly pedestrians, and all pedestrians per Madrid district for the period 2006–2018.

Geolocation of the accidents is a key issue in this research, as accidents will be assigned to the Madrid city district they occurred in. A logical process was developed to geolocate these accidents from the available alphanumerical information about their position (i.e., street name and number or two street names if the location is a junction). It was possible to geo-locate 93% of the vehicle–pedestrian collisions. As a result, Figure 2 shows the number of collisions suffered by elderly pedestrians, non-elderly pedestrians, and all pedestrians per Madrid district for the period 2006–2018. A larger number of accidents are in the central districts in comparison to the periphery. Moreover, the figure shows that the area (surface) of districts is quite heterogeneous, and this heterogeneity is also common for other district variables, such as their road network, and other built environment indicators. Moreover, this heterogeneity is also maintained, to a greater or lesser extent, inside each district.

To assess the impact of the built environment features on elderly pedestrian road safety and compare it to impact on the group of non-elderly pedestrians, data regarding Madrid districts were gathered. According to the most used built environment variables in the literature [42,43,44], different groups of variables were collected and processed. These variables can be classified as follows: (i) socioeconomic variables, (ii) land use variables, and (iii) infrastructure features of each district. The variables of each group were selected considering the literature analysis and the availability of the data. It was especially difficult to select infrastructure variables, as there are no exhaustive inventories on Madrid city street features (e.g., width of the streets and existence and width of the sidewalks). As exposure variables, the Annual Average Daily Traffic (AADT) and total street length of each district were used. The pedestrian flow was not considered because of the lack of these data in official databases. The exposure variables are expected to contribute to the occurrence of vehicle–pedestrian collisions. Due to heterogeneity of districts, the variables were normalized to compare districts with different surfaces and street lengths. There is a big heterogeneity between the surfaces of the districts, and some of them—especially the peripheral ones—include high-capacity street (highways) and non-urban areas. As a consequence, the surface of the district seems unsuitable to normalize all the independent variables. Land use variables that deal with surfaces will be normalized by the district surface, but district surface does not provide information about the “useful area” of the districts, meaning the area where pedestrians have real interactions with the motorized traffic. Nevertheless, total street length of each district is a fair normalizing value for infrastructure variables because most of these indicators are distributed along the streets (e.g., signals, traffic lights, and street junctions).

The first group of gathered data, socioeconomic indicators (i), consists of the number of inhabitants (elderly, non-elderly, and total), population density, ageing rate, and average annual income per household in each district. The number of inhabitants of each district per year was available directly at Madrid City Council in five-year age groups. Hence, the number of the elderly, non-elderly, and total inhabitants was calculated for each district and year. These variables were normalized by total street length of the district to represent the level of exposure of each age group. Population density was also available at Madrid City Council for each district and year. Moreover, as the data about the number of inhabitants of each district were separated into age groups, the ageing rate (the proportion of inhabitants older than 65 years over the total number of inhabitants of each district) was calculated. The average annual income per household was accessible through the Madrid Council Statistical Portal for the years 2013 to 2017 and in the INE for 2015 to 2018. With respect to the rest of the years, the value of the closest available year was used.

The second group (ii), land use variables, consists of the number of Points of Interest (POIs) of a different nature per street kilometer and the proportion of a group of nominal land use indicators—residential, green area, and main street proportion. The number of POIs per kilometer in each district was considered invariant over the studied period. It was obtained through the Businesses Census published at Madrid Council Open Data Portal (MCODP), which includes leisure, retail, education centers and hospitals, among others. Finally, the number of these points was divided by the total street length of each district. Nominal land use was obtained directly from MCODP as a polygon-shaped file compatible with GIS software, containing information from the “Plan General de Ordenación Urbana de Madrid (PGOUM)”. For this study, residential and green area proportions of the total district surface were selected and considered invariable over time. Commercial use was dismissed as the variance between districts and its absolute value were almost null. Green area is defined by the PGOUM (1997) [51] as the land that provides one of these services: (i) neighborhood green area, garden area of small or medium surface, (ii) district park, gardens with a medium or large surface where people can develop multiple activities, (iii) urban park, similar to district parks but with historical or functional singularities, and (iv) metropolitan park, forest areas that offer different cultural and recreational activities that are integrated in the natural environment. The “main street” proportion was estimated according to the definition of “main street”, defined by [51] as the public road that enables the mobility and accessibility between districts due to its functional conditions, design features, traffic flow, or associated activities. Usually, main streets are arterial streets at urban level that are wider, more signalized, more illuminated, and with a higher vehicle traffic volume than the rest of the road network. The main street proportion was obtained using the nominal land use data as the ratio of main street surface over the total street surface.

The third group (iii), infrastructure, consists of those variables linked to the street type directly available from the official and open databases, such as total street length, sidewalk density (district total sidewalk surface per district street length), street junctions per kilometer, signaling per kilometer, traffic lights per kilometer, and public transport presence—bus stops and metro stations per kilometer. Total street length and street junctions per kilometer were extracted from the National Center for Geographic Information (CNIG) road axis. “Road axis” includes roads that can be used by vehicles and pedestrians simultaneously (e.g., excluding highways). Sidewalk density was obtained as the surface of sidewalk over the total street length of each district. Signaling density and traffic light density were obtained as the total number of road signals and traffic lights in a district, with their location available through the MCODP, over the total street length. Bus stops and Metro station locations and opening/closing dates were available thought the Madrid Regional Transport Consortium (CRTM) database.

Among infrastructure variables, AADT was also considered as an indicator of the infrastructure use. The AADT was only available for a group of street segments in the MCODP, so AADT was estimated for each district as the median of the traffic flow registered in the traffic gauging station located in a district. AADT is an important variable to include in this research as it shows the level of accident risk exposure of each street. This macroscopic research, at district level, forces the estimation of a medium AADT per district in the city, which can be a rude estimation mainly in those districts with high differences between traffic flows in their streets.

**Figure 3:**
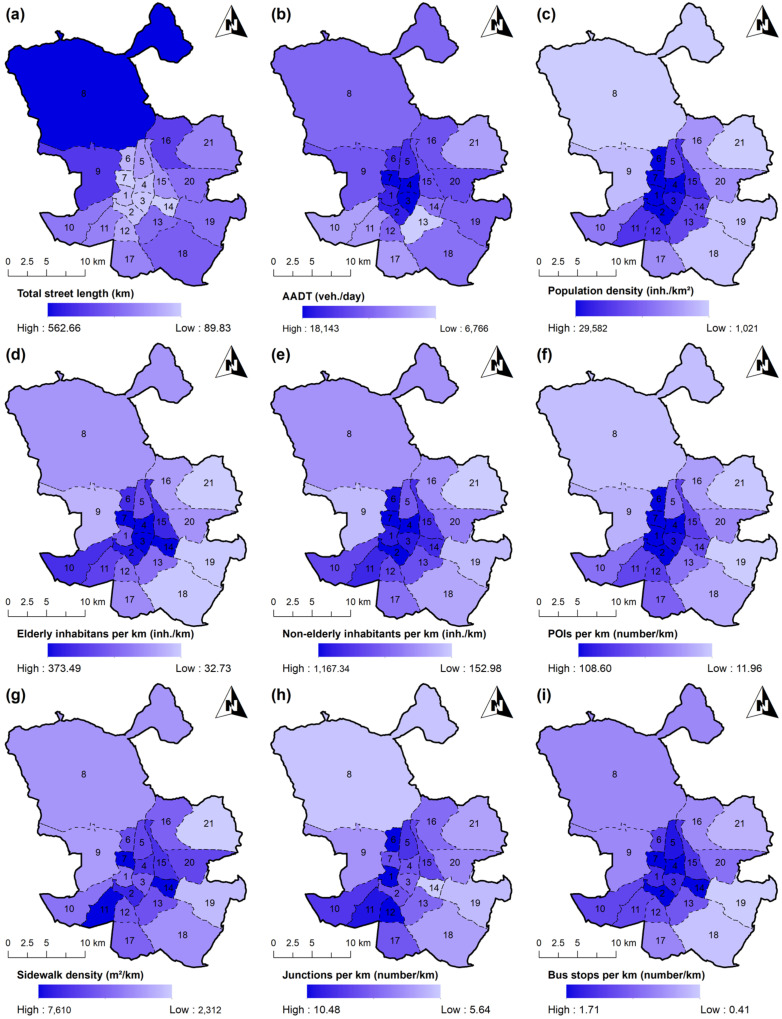
Spatial distribution of built environment variables in Madrid case study in 2018: (**a**) total street length, (**b**) AADT, (**c**) population density, (**d**) elderly inhabitants per kilometer, (**e**) Non-elderly inhabitants per kilometer, (**f**) POIs per kilometer, (**g**) sidewalk density, (**h**) junctions per kilometer, and (**i**) bus stops per kilometer.

The result of this process was an ad hoc-designed database that contains different built environment indicators of Madrid as a case study. In order to show district heterogeneity, this information can be displayed on a map to study the spatial distribution of the variables. Figure 3 shows graphically a subset of the gathered variables on a Madrid districts map. The total street length is higher in bigger peripheral districts, mainly in Fuencarral-El Pardo (8), Moncloa-Aravaca (9), Hortaleza (16), Villa de Vallecas (18), and Puente de Vallecas (13). In terms of traffic, AADT is greater in central districts such as Retiro (3), Salamanca (4), and Chamberí (7), where there is a higher concentration of POIs. Nevertheless, in this study AADT was obtained as the median value of a group of measuring points provided by Madrid Council. Hence, this variable is only an average measure of a district traffic flow as gauging stations are usually located in main streets and not in narrower residential ones. In relation to population density, values are larger in the city center districts, those that have a smaller surface area, especially in Chamberí (7), Tetuán (6), Salamanca (4), and Centro (1), which are the four most centrally located districts. Elderly and non-elderly inhabitants per total street length have a similar behavior, being maximum in Chamberí (7), Retiro (3), Salamanca (4), and Moratalaz (14) for the elderly pedestrians, and in Chamberí (7), Arganzuela (2), Salamanca (4), and Tetuán (6) for the non-elderly pedestrians. If we focus on land use variables, POIs per kilometer have the same distribution as that of population density. This variable also represents an attraction motivation for inhabitants of other districts to visit, being this indicator higher in Centro (1), Chamberí (7), Arganzuela (2), Salamanca (4), and Tetuán (6). Sidewalk density is higher in those districts with wider sidewalks or sidewalks all along the streets. In fact, the sidewalk density is an average indirect measurement of the sidewalk width. The lack of sidewalks in some streets reduces the value of this indicator even though the existent ones are wider than those in other districts. Figure 3 shows that this variable does not follow a spatial pattern of distribution among districts as happens with previous variables. However, sidewalk is higher in districts located in a central north–south band, especially the suburban districts, being remarkably higher in Moratalaz (14), Chamberí (7), and Carabanchel (11). The indicator “junctions per kilometer” is higher in districts with intricate road networks. This fact applies mainly in central and south districts, especially in Centro (1), Tetuán (6), Usera (12), and Carabanchel (11). Finally, “bus stops per kilometer” is higher in central districts, as these are attraction areas and, as a consequence, the public transport network is denser. This indicator is also related with population density and inhabitants per kilometer variables, being maximum in Moratalaz (14), Chamberí (7), Arganzuela (2), and Salamanca (4).

### 3.2. Methodology

The main objective of this paper was to assess the influence of the built environment of a city on the occurrence of vehicle–pedestrian collisions considering the ageing of the population. This analysis was developed at the macroscopic level, using Madrid districts as the spatial unit of reference. District level was selected with the aim to use mainly the data available in official databases that did not require a big amount of postprocessing operations, and the lowest level of detail found in these data was the district level. Once a database containing the dependent (number of accidents) and independent (built environment features) variables was obtained, two regression models were formulated and compared, using all the studied contribution factors: one model for vehicle–pedestrian collisions where the pedestrian was 65 or older, and other for the rest of vehicle–pedestrian crashes. The purpose was to evaluate the statistical significance and sign of each variable used in the models and study the differences between them. For this objective, the statistical regression model employed was the negative binomial regression, a widely used model on random discrete events with overdispersion. In this section, the construction of the two negative binomial statistical models is described, using the ad hoc-designed datasets explained in the previous section.

The Negative Binomial (NB) distribution is a very frequently employed regression model in studies on the occurrence of road crashes of different natures, including studies at a macroscopic level [52]. The NB is a distribution derived from the Poisson gamma distribution [53], and it was employed because it can operate with data with overdispersion. This feature cannot be found in a Poisson regression model, as mean and variance are identical. Since overdispersion was found in the occurrence of pedestrian–vehicle collisions by district dataset, the NB model was appropriate to build the statistical models to be analyzed. The Probability Density Function (PDF) of the NB distribution is as follows:

(1) $$P\left(Y=y_{i}\right)=\frac{\Gamma \left(y_{i}+\alpha^{-1}\right)}{\Gamma \left(\alpha^{-1}\right)y_{i}!}{\left(\frac{\alpha \cdot \mu_{i}}{1+\alpha \cdot \mu_{i}}\right)}^{y_{i}}{\left(\frac{1}{1+\alpha \cdot \mu_{i}}\right)}^{\alpha^{-1}}$$

where P(Y = y_i_) is the probability of Y resulting in y_i_, μ_i_ is the projected number of crashes, y_i_ is the number of vehicle–pedestrian collisions at the district I, and α is the dispersion parameter.

The expected number of crashes can be calculated using the following equation:

(2) $$\mu_{i}=\exp\left(\beta_{0}+\sum_{j=1}^{n}{\beta^{\prime }}_{j}x_{\mathrm{ij}}\right)$$

where μ_i_ is the projected number of crashes, β_0_ is the intercept of the model, β_j_ are the estimated parameters of the variables, and x_ij_ are the independent and known variables at the district i.

**Figure 4:**
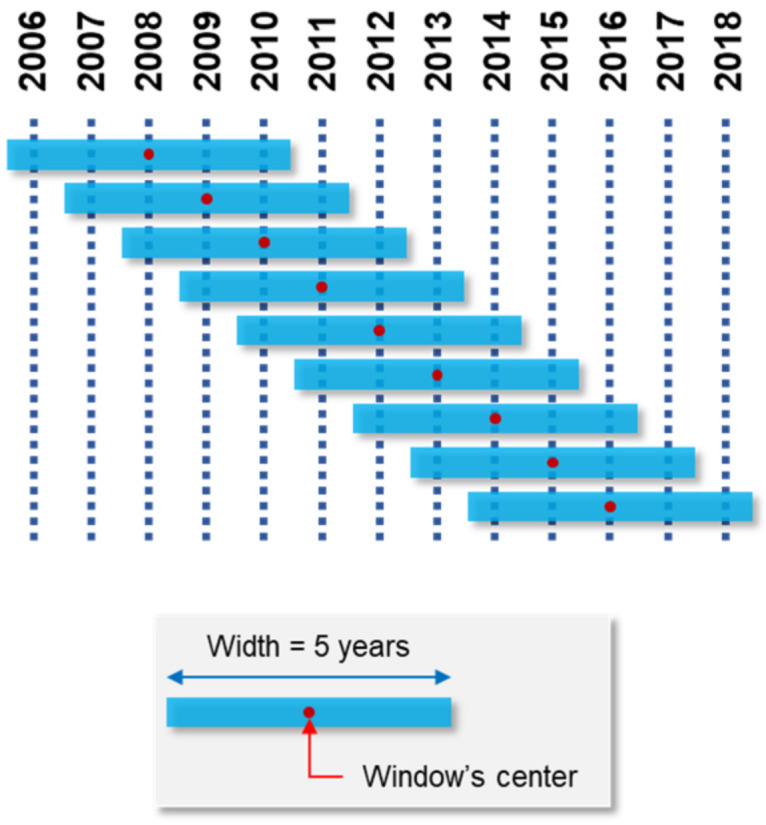
Sliding window method applied to Madrid case study in the period 2006–2018.

As accident occurrence is a random event and the studied independent variables of Madrid districts do not vary greatly, the input of the negative binomial model had to be treated due to the variance of the number of vehicle–pedestrian crashes in consecutive years of the study. With this approach, the Sliding Window Method (SWM) was used in the data. The SWM consists of “moving” a virtual window of a specified width over a tidy database to obtain the result of a certain function (e.g., sum, average, or median value) applied to a variable in the range covered by the window [54,55]. A five-year window was used in this investigation, and, as the period of study was 13 years, nine complete windows were created for each city district (see Figure 4). As a consequence, nine records of each district (21 in total) were available, summing up a total of 189 observations. On the one hand, the number of vehicle–pedestrian collisions (the dependent variable) in each five-year window was calculated as the sum of the events that occurred during that period. Thus, the output variable of the model is “number of vehicle–pedestrian collisions in 5 years in the district”. On the other hand, for the independent variables (socioeconomic, land use, and infrastructure), the used function was the average value for each five-year period.

At this point, dependent and independent variables were already collected and processed. Table 1 shows the main statistics associated to the built environmental variables classified in three groups (socioeconomic, land use, and infrastructure). Thus, the Negative binomial regression model was applied, and two models, using all the studied variables, were formulated in order to compare results: one model for vehicle–pedestrian collisions where the pedestrian was 65 or older, and other for the rest of vehicle–pedestrian crashes. In both models, two exposure variables were used: total street length and AADT (Equation (3)).

(3) $$\mathrm{Acc}=L^{\beta_{1}}\cdot {\mathrm{AADT}}^{\beta_{2}}\cdot \exp\left(\beta_{0}+\beta_{3}\cdot \mathrm{InhExp}+\beta_{4}\cdot \mathrm{Pop}\;D+\beta_{5}\cdot \mathrm{AI}+\beta_{6}\cdot \mathrm{POIs}+\beta_{7}\cdot R\;\mathrm{Prop}+\beta_{8}\cdot G\;\mathrm{Prop}+\beta_{9}\;\cdot \mathrm{MS}\;\mathrm{Prop}+\beta_{10}\cdot \mathrm{Swk}\;D+\beta_{11}\cdot \mathrm{Junct}+\beta_{12}\cdot \mathrm{Signals}+\beta_{13}\cdot \mathrm{TLights}+\beta_{14}\cdot \mathrm{Metro}+\beta_{15}\cdot \mathrm{Bus}\right)$$

where “Acc” is the number of vehicle–pedestrian collisions, where pedestrians are elderly or non-elderly depending on the model, during a five-year window in a district, “L” is the total district street length (km), “AADT” is the average AADT per district (veh./day), “InhExp” is the number of inhabitants of the studied age group (elderly or non-elderly, depending on the model) per kilometer (inhabitants/km), “Pop D” is population density (inhabitants/km^2^), “AI” is average annual income per household (€), “POIs” is POIs per street kilometer (points/km), “R Prop” is residential proportion (% of the district surface), “G Prop” is green area proportion (% of the district surface), “MS Prop” is main street proportion (% of the street surface over the district surface), “Swk D” is sidewalk density (m^2^/km), “Junction” is the number of street junctions per street kilometer (junctions/km), “Signals” is the number of signals per street kilometer (signals/km), “Tlights” is the number of traffic lights per street kilometer (traffic lights/km), “Metro” is the number of metro stations per street kilometer (metro stations/km), and “Bus” is the number of bus stops per street kilometer (bus stops/km).

## 4. Modelling Results

Table 2 shows the results of the modelling, the level of significance of each independent variable, the Akaike Information Criterion (AIC) and the log-likelihood of the models. Differences between both models have been studied through the statistical significance (*p*-value) of the independent variables and the sign (positive or negative) of the parameters.

Two exposure or control variables were considered: total street length and AADT of the district. The first one, total street length, is significant in both models and has been included in the models to distinguish two districts with the rest of the variables being equal. As expected, this indicator has a negative effect on road safety, as higher total street length involves more locations at which to suffer an accident. Despite that, this macroscopic analysis does not consider if the streets “could not be crossed”, understanding the formal allowance of crossing a certain road through the existence of zebra crossing and the real allowance of crossing a certain road based on the width, traffic flow, and type of the road. Ring roads linked to big cities are usually located at peripheral districts, and this regional approach does not allow a deeper level of analysis. This way, each street of a certain district is thought to have the same accident risk exposure, regardless of the differences between them in terms of infrastructure design, and this fact is crucial in heterogeneous districts. The research also did not consider if streets have at least a sidewalk on one of its sides. The second exposure variable, AADT, shows that elderly pedestrian collisions are much more conditioned by AADT than younger-pedestrian collisions are, and it has a positive impact (positive sign) in both models, as it is a measure of accident risk exposure. This result is consistent, since the elderly have a longer reaction time, and a high traffic volume involves the reduction of time between vehicles (i.e., the spatial and temporal window gap to cross a street). Nevertheless, in this study, AADT is estimated as an average value per district, and the study does not consider if this traffic is registered in roads that are not “walkable” (clearly with an urban topology, provided with suitable crossing areas).

Regarding socioeconomic variables, both models show that population density has a positive and significant effect on the number of collisions, with the significance of the ageing rate being higher in model 2. Population density collects how crowded a district is, considering the average height of buildings, and usually it represents a measure of pedestrian accident risk exposure. The number of elderly inhabitants per total street length has a positive effect on the number of elderly pedestrian collisions (model 1), while the number of non-elderly inhabitants per kilometer is not statistically significant for non-elderly casualties (model 2). This fact is consistent with the idea that elderly pedestrians are likely to walk within their neighborhood/district of residence while younger people usually move towards crowded and central areas. Annual average income is an indicator of the economic status of a district, and as it increases, the likelihood of the number of collisions is reduced. Both models reflect this effect, and this variable is significant in both models.

In relation to land use variables, model 2 shows a high effect of POIs per street length, residential proportion, and green area proportion on the occurrence of non-elderly pedestrian collisions. In that model, more residential proportion means less risk for non-elderly pedestrians. In relation to Points of Interest (POIs) per street length, a higher density means less risky areas for non-elderly pedestrians. This result was not a priori expected but deals with the idea that a higher density of POIs indicates more crowded streets where pedestrians walk slower and are more alert to their surroundings. POIs per street length, residential proportion, and green area proportion are statistically insignificant for the occurrence of elderly pedestrian collisions (model 1). Finally, main street proportion is an indicator of the importance of the streets within a certain district. Usually, these streets are wider and have higher traffic flows operating at higher speeds compared with the rest of streets. A higher proportion of these streets represents a higher level of accident risk exposure for pedestrians in both models (regardless of their age).

Concerning infrastructure variables, both models show a high significance of junction density and bus stop density. Sidewalk density has a negative effect on the number of accidents, and this effect is only statistically significant in the case of elderly pedestrians. Sidewalk density is an indicator of the amount of sidewalk available for the pedestrian in relation to total street length. In other words, it represents an average width of the sidewalk, being narrower in central districts and wider in the newer districts. Elderly pedestrians are more sensitive to the state and the width of the sidewalk, as this population group has a higher risk of falling, stumbling, or stripping while walking compared with younger adults. For the elderly, maintaining postural stability and balance is more difficult [8], and usually too narrow sidewalks incite pedestrians to walk outside the sidewalk [18]. This is a riskier task for the elderly, as inadequate infrastructures can increase elderly pedestrians’ anxieties on the perception of the built environment [16,17]. Results also indicate that a higher number of street junctions per kilometer favors the number of collisions in one district for both elderly and younger pedestrians, as this variable represents how complex and intricate a street network is and, in consequence, how many hazards a pedestrian or driver can find for the same total street length. The density of traffic lights significantly improves road safety for elderly pedestrians, while it is statistically insignificant for the rest of pedestrians. The presence of traffic lights implies a lower speed of the traffic flows as vehicles are forced to stop when traffic lights are red. The statistical insignificance of this variable in the non-elderly pedestrian model suggests a higher level of awareness of the elderly. In relation with public transport, bus stop density is an indicator of riskier districts for both elderly and non-elderly pedestrians. The density of bus stops has a positive effect on the number of accidents, as it represents a possible higher pedestrian flow, the presence of large vehicles in the streets, and the existence of critical spots where the elderly can mislead when getting off the bus. Metro station density, which is a measure of generation and attraction of pedestrian trips of a certain zone, has also a positive effect, but it is only statistically significant for non-elderly pedestrians (model 2), reinforcing the idea that non-elderly pedestrians are mobile over wider zones than the surrounding of the district of residence of the pedestrian and that this age group moves towards central areas. In summary, among all the infrastructure variables analyzed, there are two that significantly affect the road safety of the elderly pedestrian: the average sidewalk width and traffic lights per kilometer.

In conclusion, the comparison of the two models reveals that districts with higher total street length, population density, “main street” proportion, street junctions per kilometer, and bus stops per kilometer and lower average income are riskier for pedestrians in general, regardless their age. In particular, the occurrence of elderly pedestrian collisions (model 1), unlike younger pedestrian accidents, is favored by traffic flow (AADT) levels and the population of the age group per kilometer, while these variables are statistically not significant in the occurrence of non-elderly pedestrian casualties. In turn, accident occurrence is less likely with a higher sidewalk density and more traffic lights per kilometer. Non-elderly pedestrian collisions (model 2) are favored by greater signals density and metro stations per kilometer, and are less affected by AADT levels.

The results of this study lead us to think that the general actions aimed at reducing the road accident rate of pedestrians are not going to be enough to drastically decrease the number of fatal and severe elderly pedestrian collisions. There is a scientific consensus on the need to improve street connectivity for all road users, for well-designed sidewalks in all streets, tighter intersection turning radii, bicycle facilities, and the use of two-lane streets whenever possible. However, the results of this paper show that neighborhoods with a higher number of elderly inhabitants per kilometer are going to need specific actions, apart from those previously mentioned. These actions should not only concentrate on “over-marking” (and “over-signaling”) crosswalks and intersections in these districts or reinforcing the use of “traffic calming” measures. Pedestrian facilities near transit stations also require special attention in those ageing districts as the interaction with traffic flows is higher and the perception and reaction times of pedestrians increase with the physical deterioration derived from ageing. In conclusion, a special package of countermeasures is needed for the elderly if policymakers aim to achieve the design of more sustainable “age-friendly” cities.

## 5. Conclusions and Discussion

The current ageing process of the population is a matter of concern in almost every scientific field of knowledge. This paper assessed this issue from the urban road safety perspective, considering vehicle–pedestrian collisions in Madrid city as a case study. Elderly pedestrians show a higher intrinsic fragility when they suffer an accident due to their physical and mental limitations caused by age-related illnesses and certain chronic medication. These limitations are also connected to a higher crash risk. Previous literature has shown behavior peculiarities of the elderly as pedestrians, such as acting more cautiously because of their maturity or avoiding complex traffic situations. The study of this age group, particularly acting as pedestrians, will be suitable to prepare the built environmental factors and adapt our cities for the future with a higher rate of aged population.

In this research, data per Madrid district were gathered to build two negative binomial regressions in order to estimate the number of vehicle–pedestrian (elderly and non-elderly) collisions considering built environment indicators. For this purpose, an ad hoc dataset containing variables at Madrid district level per year was constructed and treated using the sliding window method with five-year width windows. Mainly primary data were gathered, available directly from official databases that did not require a big amount of postprocessing operations. The dependent variable of the models was the number of vehicle–pedestrian collisions per district during each five-year sliding window for the total studied period, 2006–2018.

The main results show that elderly pedestrian collisions are favored by traffic flow (AADT) levels and the population of the age group per kilometer, while these variables are statistically not significant in the occurrence of non-elderly pedestrian casualties. On the other hand, elderly collisions are less likely with a higher sidewalk density and more traffic lights per kilometer. These results show that countermeasures based only on improving infrastructure (signaling of crosswalks and intersections, implementing crossing islands, and well-designed sidewalks) will help to reduce pedestrian collisions but will not be enough to reduce specifically elderly pedestrian collisions. As the level of road safety risk exposure of the residence district is dependent on population density and AADT as determinant factors, additional actions will have to be implemented specifically in districts with an aged population. Most of them should have the objective to improve the conspicuity of elderly pedestrians for drivers and specifically improve the perception of elderly pedestrians about other road users in these districts. For example, apart from reducing vehicle flow speeds, narrowing street lanes, and widening sidewalks, new traffic signs with elderly pedestrian symbols could be implemented.

Despite the results obtained in this investigation, new research lines emerge, as improvements in the methodology can be achieved. First, the sample size was conditioned by the level of detail of the information available at official data sources but could be widened in the future. Moreover, the study at district level may lead to confusing results because this spatial unit is not found to be homogenous itself, and this problem could be solved by studying the phenomenon using a smaller spatial unit, such as neighborhoods, or using regular grids. Consequently, a more complex and ad hoc data collection process would be needed to feed the new territorial divisions. If using regular grids, instead of neighborhoods, the size of the cells of the grid would be unknown a priori. Thus, a comparative analysis between a set of possible sizes would also be required. Finally, the elderly pedestrian group could be compared to other groups with physical limitations, such as disabled people, to analyze potential common measures to improve road safety of both groups in urban areas.

A second research line can be oriented to study groups of street segments with the same typology and traffic flow. This line represents a major challenge in the already used methodology, and it is a common tool in the study of accident black spots (high-accident-concentration sections) in interurban roads. It is considered to be a correct alternative, as it will be possible to find and classify street segments based on built environment features and the existence or absence of vehicle–pedestrian collisions. The street segments approach would need a deeper definition of variables with an ad hoc measurement of infrastructure indicators (e.g., the sidewalks width). Despite the opening of new research lines, one of the main challenges of the study of urban accidents, the geolocation of the accidents, was properly solved in this paper by developing an ad hoc procedure. The validation of this procedure in this Madrid case study will be valuable in further investigations, regardless of the research line followed.

The need to obtain a more complete dataset on infrastructure variables is a common issue in future research lines and, as this study has demonstrated, is also a key point (among the rest of built environment variables) to achieve a holistic understanding of elderly pedestrian collisions. Transport policymakers can take actions on the city infrastructure, and the final goal of this research is to define scientifically a set of rules and recommendations to build or redesign sustainable cities considering the ageing process of the population, thus reducing the risk of elderly pedestrian collisions.

## Figures and Tables

**Table 1:** Main statistics of the built environment variables considered in the research by type (land use, socioeconomic, and infrastructure).

Variable	Unit	Min.	Max.	Mean	Median	σ (SD)
**Exposure**						
Total street length (L)	km	89.83	562.66	243.54	252.82	123.28
AADT (AADT)	veh./day	6766.00	18,143.00	11,299.00	11,052.00	2727.46
**Socioeconomics**						
Elderly inhabitants per km (InhExp)	inh./km	18.80	378.02	157.24	157.22	92.35
Non-elderly inhabitants per km (InhExp)	inh./km	148.00	1298.70	622.70	642.00	298.14
Population density (Pop D)	inh./km^2^	880.00	32,227.00	14,263.00	15,822.00	9726.10
Average income per household (AI)	€/district	23,517.00	70,735.00	38,456.00	35,532.00	10,992.18
**Land use**						
POIs per km (POIs)	points/km	11.96	108.60	49.14	43.09	28.17
Residential proportion (R Prop)	% Surface	0.03	0.48	0.27	0.28	0.14
Green area proportion (G Prop)	% Surface	0.00	0.41	0.08	0.05	0.09
Main street proportion (MS Prop)	% Surface	0.28	0.68	0.46	0.46	0.10
**Infrastructure**						
Sidewalk density (Swk D)	m^2^/km	2312.00	7610.00	4702.00	4588.00	1370.84
Junctions per km (Junct)	junctions/km	5.64	10.48	7.59	7.68	1.27
Signals per km (Signals)	signals/km	14.47	83.05	42.95	40.41	16.35
Traffic lights per km (TLights)	lights/km	1.62	21.02	9.16	8.10	4.83
Metro stations per km (Metro)	stations/km	0.00	0.36	0.08	0.04	0.09
Bus stops per km (Bus)	stops/km	0.26	1.71	1.01	1.05	0.39

**Table 2:** Results of the Negative Binomial (NB) regression models.

	Model 1: Elderly Pedestrians				Model 1 and Model 2 Comparison		Model 2: Other Pedestrians			
Variable	Estimate	S. Error	z Value		Sign	*p*-Value	Estimate	S. Error	z Value	
**Intercept**	−1.49 × 10^−1^	1.21	−12.321	***	=	<	−8.15	9.50 × 10^−1^	−8.574	***
**Exposure**										
log (Total street length)	2.00	1.10 × 10^−1^	18.199	***	=	<	1.55	9.92 × 10^−2^	15.659	***
log (AADT)	5.73 × 10^−1^	1.14 × 10^−1^	5.032	***	=	<	1.26 × 10^−1^	8.08 × 10^−2^	1.563	
**Socioeconomics**										
Inhabitants per km	2.74 × 10^−3^	3.20 × 10^−4^	8.572	***	=	<	5.80 × 10^−5^	2.01 × 10^−4^	0.289	
Population density	6.01 × 10^−5^	7.48 × 10^−6^	8.036	***	=	>	8.33 × 10^−5^	7.07 × 10^−6^	11.787	***
Average income	−2.42 × 10^−5^	2.50 × 10^−6^	−9.661	***	=	<	−1.66 × 10^−5^	1.92 × 10^−6^	−8.624	***
**Land use**										
POIs per km	2.42 × 10^−3^	3.49 × 10^−3^	0.693		≠	>	−1.20 × 10^−2^	3.42 × 10^−3^	−3.500	***
Residential proportion	9.26 × 10^−1^	5.43 × 10^−1^	1.705	.	≠	>	−1.66	4.47 × 10^−1^	−3.705	***
Green area proportion	5.98 × 10^−2^	1.54 × 10^−1^	0.388		=	>	8.20 × 10^−1^	1.44 × 10^−1^	5.691	***
Main street proportion	2.04	4.59 × 10^−1^	4.436	***	=	>	2.04	3.58 × 10^−1^	5.692	***
**Infrastructure**										
Sidewalk density	−8.26 × 10^−5^	1.57 × 10^−5^	−5.276	***	=	<	−1.39 × 10^−5^	1.38 × 10^−5^	−1.010	
Junctions per km	8.15 × 10^−2^	2.65 × 10^−2^	3.078	**	=	>	2.18 × 10^−1^	2.40 × 10^−2^	9.078	***
Signals per km	−1.42 × 10^−3^	2.92 × 10^−3^	−0.488		≠	>	1.17 × 10^−2^	2.67 × 10^−3^	4.389	***
Traffic lights per km	−2.50 × 10^−2^	8.66 × 10^−3^	−2.889	**	≠	<	7.73 × 10^−3^	8.68 × 10^−3^	0.891	
Metro stations per km	3.87 × 10^−1^	4.20 × 10^−1^	0.920		=	>	2.65	3.55 × 10^−1^	7.465	***
Bus stops per km	1.42	1.20 × 10^−1^	11.820	***	=	<	9.41 × 10^−1^	1.28 × 10^−1^	7.367	***
**Number of observations (*n*)**	189						189			
**AIC**	1444.2						1713.6			
**Log-likelihood**	−705.1						−839.8			

Significance codes (*p*-value): ‘***’ for 0.1%, ‘**’ for 1%, ‘*’ for 5%, and ‘.’ for 10%.

## Data Availability

The data presented in this study are available on request from the corresponding author.
